# *Adonis amurensis* Is a Promising Alternative to *Haematococcus* as a Resource for Natural Esterified (3*S*,3′*S*)-Astaxanthin Production

**DOI:** 10.3390/plants10061059

**Published:** 2021-05-25

**Authors:** Yongfu Li, Fengying Gong, Shuju Guo, Wenjie Yu, Jianguo Liu

**Affiliations:** 1 CAS Key Laboratory of Experimental Marine Biology, Center for Ocean Mega-Science, Institute of Oceanology, Chinese Academy of Sciences, Qingdao 266071, China; liyongfu@qdio.ac.cn (Y.L.); zhanglitao@qdio.ac.cn (F.G.); guoshuju@qdio.ac.cn (S.G.); yuwenjie@qdio.ac.cn (W.Y.); 2 Laboratory for Marine Biology and Biotechnology, Qingdao National Laboratory for Marine Science and Technology, Qingdao 266071, China; 3 University of Chinese Academy of Sciences, Beijing 100049, China

**Keywords:** astaxanthin, *Adonis amurensis*, geometric isomers, optical isomers, pigment distribution

## Abstract

Astaxanthin (AST) characteristics and pigment productivity of *Adonis amurensis*, one of the few AST-producing higher plants, have not yet been studied extensively. In this study, the geometrical and optical isomers of AST in different parts of the *A. amurensis* flower were determined in detail, followed by a separation of the all-*trans* AST using HPLC chromatography. AST extracted from the flower accounted for 1.31% of the dry weight (dw) and mainly existed in the di-esterified form (>86.5%). The highest concentration was found in the upper red part of the petal (3.31% dw). One optical isomer (3*S*, 3′*S*) of AST, with five geometrical isomers (all-*trans*, 9-*cis*, 13-*cis*, 15-*cis*, and di-*cis*) were observed in all parts of the flower. All-*trans* AST was the predominant geometrical isomer accounting for 72.5% of the total content of geometric isomers in total flower, followed by the 13-*cis*, and 9-*cis* isomers. The all-*trans* AST isomer was also isolated, and then purified by HPLC from the crude oily flower extract, with a 21.5% recovery yield. The *cis*-AST extracted from the combined androecium and gynoecium gives a very strong absorption in the UVA region due to a high level of *cis*, especially di-*cis*, isomers, suggesting a prospective use in the preparation of anti-ultraviolet agents. The production cost of AST from *Adonis* flowers can be as low as €388–393/kg. These observations together with other factors such as the low technology requirement for plant culturing and harvesting suggest *Adonis* has great potential as a resource for natural esterified (3*S*,3′*S*)-AST production when compared with *Haematococcus* culturing.

## 1. Introduction

Astaxanthin (AST), a pigment of commercial interest, imparts an attractive blue to reddish hue to the feathers, carapace, skin, and flesh of many animals, such as flamingo, shrimp, krill, crabs, salmon, and red snapper [1,2,3,4]. This lipid-soluble pigment has substantial economic value as a nutraceutical, feed additive for aquatic animals, and potential source of new pharmaceuticals for the treatment of diseases caused by oxidative stress [3,4,5,6]. AST-containing products are increasingly popular as human health food supplements, with a market size of over US$100 million in 2018, with double-figure annual growth rates [7]. These supplements are taken for many different reasons, including improving eye health and vision, skin health, and enhancing athletic performance by speeding muscle recovery after exercise. The global market for AST is rapidly increasing; however, the bulk of AST available is produced synthetically, providing an opportunity for marketing a natural product [8,9].

**Figure 1:**
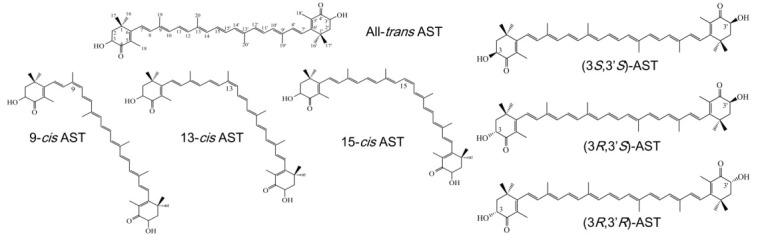
Structures of geometric and optical isomers of astaxanthin (AST).

The de novo biosynthesis of AST occurs in certain unicellular algae, some bacteria, and fungi as well as *Adonis* flowers. So far, natural AST mainly comes from *Haematococcus pluvialis* (which may be better named *H. lacustris* [10]), shrimps, the bacterium *Paracoccus* spp. and red yeast *Xanthophyllomyces dendrorhous* (also known as *Pfaffia rhodozyma*) [3,11,12,13,14]. It was reported that the current commercialized (3*S*,3′*S*)-astaxanthin used as a fish additive is mainly produced by *P. carotinifaciens* [8]. More recently, people also have tried to engineer AST-producing plants by introducing genes from bacterial and algal pathways [15,16,17], although successful mass production has not yet been achieved. In addition to *H. pluvialis*, other microalgal species such as the *Coelastrella* spp. [18,19,20], *Bracteacoccus aggregatus* [20], *Chromochloris zofingiensis* [21,22] also produce varying amounts of astaxanthin. ASTs from different sources appear as different stereoisomeric forms, which show different physiological activities [2,23,24,25]. Astaxanthin has a number of isomers on the basis of its chemical structure. The many conjugated double bonds in the polyene chain allow astaxanthin to exist as several geometric isomers, mainly the all-trans, 9-*cis*, 13-*cis*, and 15-*cis* isomers (Figure 1). AST also has two identical asymmetric carbon atoms at the C-3 and C-3′ positions, making possible three stereoisomers, including a pair of enantiomers (3*S*,3′*S*), (3*R*,3′*R*), and a meso form (3*R*,3′*S*) (Figure 1). AST can be esterified in one or both hydroxyl groups with different fatty acids such as palmitic, oleic, estearic, or linoleic: it may also be found free, that is, with the hydroxyl groups without esterification; or else, forming a chemical complex with proteins (carotenoproteins) or lipoproteins (carotenolipoproteins) [26]. The natural functions of AST are determined by their physicochemical properties which depend on their molecular structure. Previous studies have suggested that *cis*-AST, especially 9-*cis*, have a higher antioxidant activity than the all-*trans* isomer [5,26,27]. Carotenoids react rapidly with free radicals and their reactivity depends on the length of the polyene system and the terminal rings. The presence of the hydroxyl (OH) and keto (C=O) moieties on each ionone ring contributes to its high antioxidant activity [28]. Based on this view, esterified AST is more stable than free one. In synthetic AST, the ratio of (3*S*,3′*S*): (3*S*,3′*R*): (3*R*,3′*R*) isomers is about 1:2:1 [29]. In wild shrimps, the proportions of the (3*S*,3′*R*)- and (3*R*,3′*R*)-isomers to the total AST ranged from 38.85–52.01% and 14.07–29.89%, respectively [23]. In *H. pluvialis*, the (3*S*,3′*S*)-isomer is the predominant form; while that in the yeast is the (3*R*,3′*R*) form [29,30]. Therefore, there is a challenge, that is, pure (3*S*,3′*S*)-AST can only be obtained from cultured *Haematococcus* unless it can be separated from the synthetic product or krill oil. AST accumulation in *Haematococcus* cells is greatly affected by light intensity, temperature, and nutritional stresses, especially under the most frequently adopted photoautotrophic culture method [11,31]. It is generally believed that a location with high solar radiation and high temperatures is most suitable for AST production [32]. Furthermore, AST production by *H. pluvialis* is a highly skill-intensive industry, which cannot be popularized in a decentralized manner. It is unlikely that the cultivation of this alga could be performed by untrained farmers.

Red-flowered *Adonis* species are the only AST-producing higher plants [33]. Different from the culture of *H. pluvialis*, *Adonis* farming can be performed readily by farmers, based on their extensive experience growing other crops. Most species of *Adonis* grow best in moist soil with plenty of humus. Large-scale Adonis farming can be conducted in the traditional solar greenhouse covered by a transparent plastic film. Mature agricultural cultivation experience, especially in agricultural greenhouse cultivation, is sufficient for the cultivation of *Adonis* [34,35]. The cultivation of this plant has the advantages of requiring only easy, relatively low-cost, farming technology, making it a low-risk investment that is easy to decentralize [36]. So far, the biosynthetic pathway of astaxanthin in *A. aestivalis* flowers has been elucidated [37,38]. The carotenoids and their fatty acid esters in the petals of *A. aestivalis* were also identified [39]. The amount of astaxanthin that accumulates in the flower petals of *A. annua* is reported to be about 1% of the dry weight [40]. However, existing reports have focused on the extraction methods [40,41,42,43], biosynthetic pathway analysis [37,38], and product quality improvement [39,44] of AST in *Adonis*. Up to now, however, no report on the distribution and characteristics of AST and its isomers in different parts of the *Adonis* flower has been reported.

We extracted the AST from different parts of the *A. amurensis* flower, and then, analyzed the extracts using UV-visible spectroscopy and high-performance liquid chromatography (HPLC) methods. Related carotenoid substances and AST compositions, i.e., mono/di-ester and geometrical/optical isomers, were also identified and quantified, followed by purification of the all-*trans* isomer from the crude extracts of the total flower. Based on the test data, we further compared the feasibility of natural AST production comparing the microalga *H. pluvialis* and *A. amurensis*. To our knowledge, this is the first time AST production based on the quality of AST in *A. amurensis* flowers has been discussed. We expect that these data will help encourage high-yield production of AST from this plant.

## 2. Results

### 2.1. UV-Visible Absorption Spectra of Acetone Extracts

**Figure 2:**
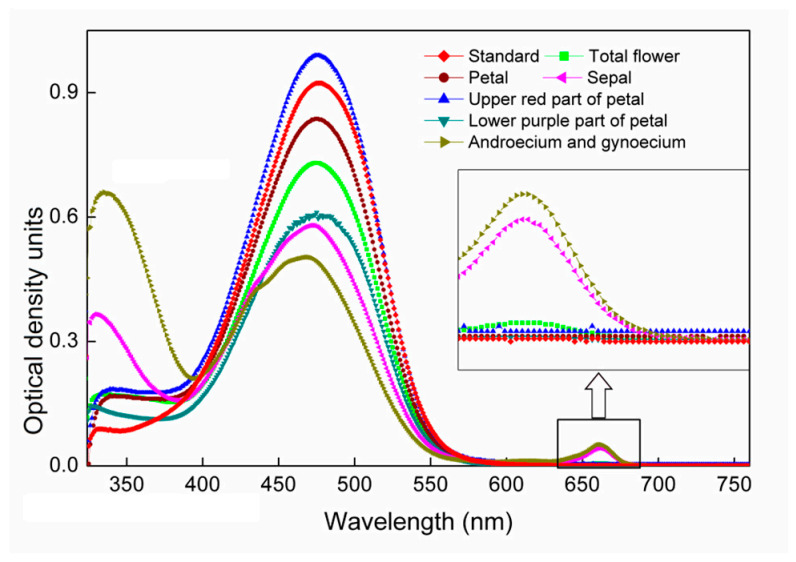
Spectrogram of pigment extracted from different parts of *Adonis amurensis* flower using pure acetone.

Lipid-soluble pigments from *A. amurensis* have a visible absorption spectrum in acetone similar to that of the AST standard, with a maximum absorption wavelength (λ_max_) of 480 nm (Figure 2). Peaks at 665 nm, observed in the spectra of the sepal and the combined androecium and gynoecium, indicated the presence of a trace of Chl *a* in these parts of the flower. Extracts of the flower, especially the androecium and gynoecium and the sepal, also showed a peak around 330–340 nm, the specific peak for *cis*- isomers of carotenoids. Inflections in the blue region in the gynoecium and androecium and sepal fractions can also be observed.

### 2.2. Pigment Contents of Flower Parts

Different flower parts showed a similar level of moisture, which ranged from 67.70% to 73.85% (Table 1). The highest carotenoid content was obtained from the red part of the petal (3.31%); in contrast, the sepal and the combined androecium and gynoecium had significantly lower pigment levels than the other parts. Chl *a* content of both the sepal and the combined androecium and gynoecium was relatively higher than that in other flower parts. Chl *b* content was lower than that of Chl *a* in total flower. Higher contents of Chl *b* were obtained in the upper red part of petal and sepal, with the values of 0.027% and 0.029%, respectively. Meanwhile, the contents of these two chlorophylls were both much lower than that of carotenoids.

### 2.3. AST Isomers of Flower Parts

**Figure 3:**
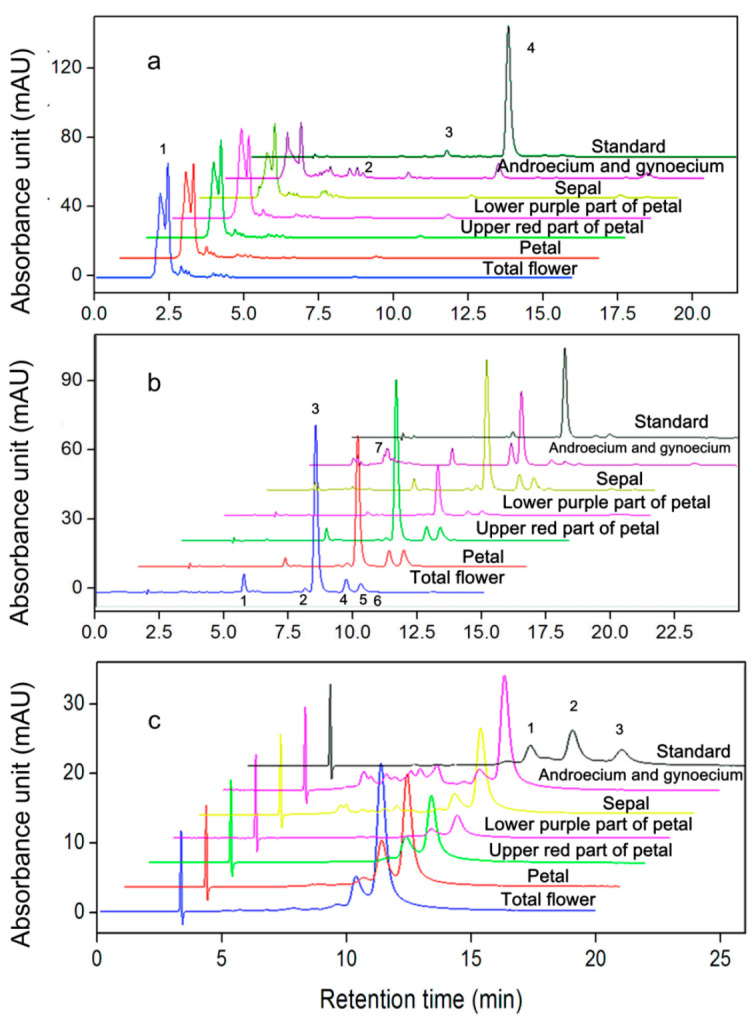
HPLC chromatograms of astaxanthin extracted from different parts of the *Adonis amurensis* flower. (**a**) Geometrical isomers of astaxanthin without enzymatic hydrolysis with all-*trans* astaxanthin as standard; (**b**) geometrical isomers of astaxanthin after enzymatic hydrolysis with all-*trans* astaxanthin as standard. HPLC condition: colum: Agilent Eclipse XDB-C18 column; mobile phase: methanol: water (95:5, *v*/*v*); flow rate: 1.0 mL min^−1^; detection: 478 nm; column temperature: 25 °C. (**c**) Optical isomer of astaxanthin and, synthesized (3*S*,3′*S*), (3*S*,3′*R*) and (3*R*,3′*R*)-astaxanthin in the ratio of 1:2:1 as standard. Astaxanthin used to detecting (**c**) were all enzymatically treated. HPLC condition: column: CHIRALPAK^®^IC column; mobile phase: methyl tertiary butyl ether: acetonitrile (35:65, *v*/*v*); flow rate: 1.0 mL min^−1^; detection: 470 nm; column temperature: 25 °C. The lines were staggered to show the isomer composition of each extract. Peaks 1, 2, 3, and 4 in (**a**) are di-ester, unknown derivatives, 13-*cis*, and all-*trans* astaxanthin respectively. In (**b**), peaks were assigned as astacene (1), di-*cis* (2), all-*trans* (3), 9-*cis* (4), 13-*cis* (5), and 15-*cis* (6) astaxanthin and β-carotene (7). In (**c**), peaks 1, 2, and 3 were identified as (3*S*,3′*S*), (3*S*,3′*R*), and (3*R*,3′*R*)-astaxanthin, respectively.

In Figure 3a, peak 4 at the retention time 8.0 min is the characteristic absorption band of all-*trans* AST. Peak 1 at 2.0–2.5 min of all plant samples was determined to be di-esterified AST, which is not present in the standard. The highest and lowest ratios of di-ester to free AST were obtained in the petal and the combined androecium and gynoecium, at 40:1 and 7:1, respectively. The chromatogram of AST after enzymatic hydrolysis (Figure 3b) showed that peaks 1–6 (retention time 5.7, 7.8, 8.5, 9.9, 10.4, and 10.9 min), represent astacene, di-*cis*, all-*trans*, 9-*cis*, 13-*cis*, and 15-*cis* AST, respectively, and these were observed in all the plant samples; however, the peak at the retention time 3.2 min (peak 7) was only observed in the spectrum for the combined androecium and gynoecium, which differed from other samples. With regard to AST optical isomers, only a peak for (3*S*, 3′*S*)-AST was found in the spectra of all samples (Figure 3c).

**Figure 4:**
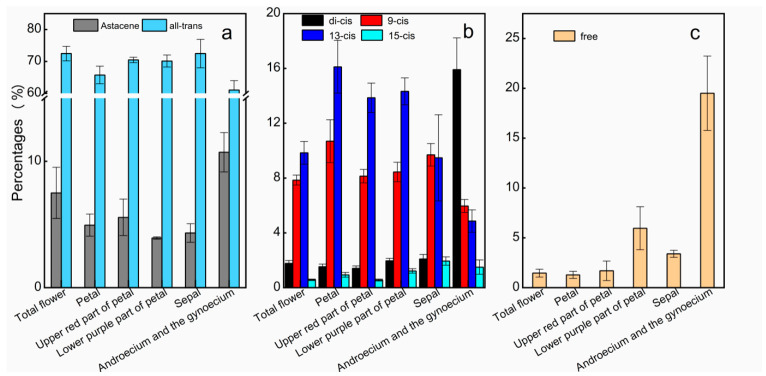
The percentages of astacene and astaxanthin in the measurable astaxanthin extracted from *Adonis amurensis* flower. (**a**) all-*trans* and astacene; (**b**) di-*cis*, 9-*cis*, 13-*cis* and di-*cis*; (**c**) free astaxanthin. Values are averages of four replicates ± standard error (SE). Eight randomly selected flowers were used to perform the determination.

The levels of different AST isomers are shown in Figure 4. Among the geometrical isomers, the all-*trans* isomer was the dominant form in *A. amurensis* flowers (about 70%). Regarding all the *cis* isomers, the proportions of the 9-*cis* and 13-*cis* isomers were significantly higher than that of 15-*cis* in the total flower. Interestingly, the levels of di-*cis* and free AST in the combined androecium and gynoecium extract were both higher than the 9-*cis*, 13-*cis*, and 15-*cis* forms, which differed from the pattern in other parts of the flower.

### 2.4. Isolation of the All-Trans Isomer of (3S,3′S)-AST

**Figure 5:**
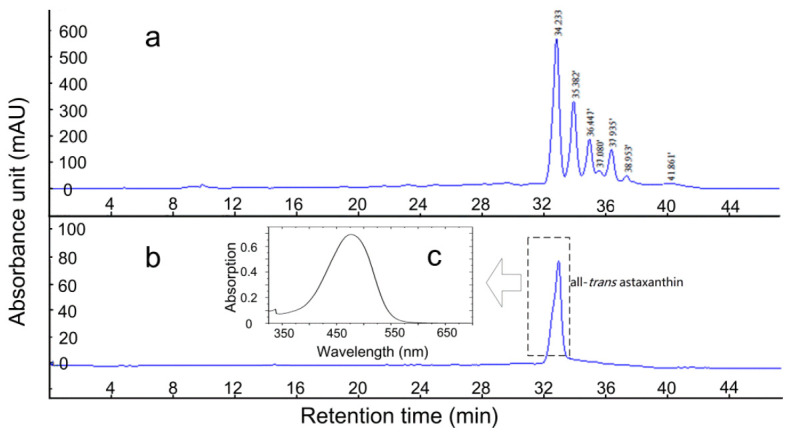
HPLC chromatograms of the crude extracts of the total flower (**a**) and the isolated all-*trans* isomer of (3*S*,3′*S*)-astaxanthin (**b**). c. the UV-visible spectrum of the purified all-*trans* isomer of 3*S*,3′*S* astaxanthin. The detection was performed at 478 nm.

In Figure 5a, the peak at the retention time of 34.23 min was confirmed to be the all-*trans* isomer of (3*S*,3′*S*)-AST. We then purified it from a crude extract of the total flower (contains 8.52% AST on the wet mass base of extract detected according to Section 4.3). The HPLC chromatograms and the UV-visible absorption spectrum of the purified all-*trans* isomer are shown in Figure 5b,c. As predicted, no peak was found in the 330–340 bands, consistent with the standard spectrum shown in Figure 1. Further calculations showed that the recovery of the all-*trans* isomer of (3*S*,3′*S*)-AST from the total carotenoids of the crude extract was 21.50 ± 0.81%.

## 3. Discussion

### 3.1. Distribution of AST and Its Isomers in Adonis Flowers

The carotenoid content (mainly AST, Figure 2) in the total flower of *A. amurensis* can be up to 1.31% (dw), found mainly in the petals. The highest AST content, 3.31%, was recorded for the upper red part of the petal, followed by the purple spot (1.21%), the sepal (0.32%), and the androecium/gynoecium (0.14%) (Table 1). The AST content of the red part of the petal resembles the content in *H. pluvialis* (commonly from 2.5% to 3.5%, dw) [45]. The AST content in the total petal is also encouraging, with a value of 2.58%.

Because free AST is relatively unstable, most AST in nature exists in the form of an ester, i.e., mono-ester or di-ester [34,46]. In *H. pluvialis* cells, approximately 70% of the AST exists as mono-esters, 25% as di-esters, and only 5% is unesterified (free) [31]. Previous studies have reported that esterified AST in cultured shrimps ranged from 47.69% to 95.56% of the total AST [23]. For *A. amurensis*, almost all of the AST exists in the di-ester form with the lowest proportion (85%) in the androecium/gynoecium and the highest level in the petal (>97.5%, Figure 3a).

AST has a broad absorption curve with a maximum wavelength of 480 nm, as shown in Figure 2. The peak at 330 nm, a characteristic of the *cis*-isomer [47,48], can be observed in the pigment’s UV-visible spectrum. Geometrical isomers of AST were further measured by using an HPLC method. The proportions of di-*cis* AST in the sepal and the androecium/gynoecium (2.10% and 15.91%, Figure 4) were both higher than in other flower parts. This result is consistent with Figure 2, in which peaks at 330 nm of the sepal and the androecium/gynoecium were significantly higher than those of other parts. However, the total *cis*-AST ratios in other parts of the flower, i.e., petal 29.3.0%, red spot 24.0%, and purple spot 25.9%, are also at the same level as that of androecium and gynoecium (23.2%) and sepal (28.2%) (Figure 4). But the intensities of the *cis* peak in the AST extracted from the petal, the upper red part of the petal, and the lower purple part of the petal are much weaker (Figure 2). High *cis* peak intensity cannot be simply attributed to the high *cis* isomer content. A previous study has shown that chlorophyll has a wide absorption band at 330 nm [49]. The relatively high content of Chl *a* in sepal and androecium and gynoecium (Table 1) may lead to higher *cis* peak intensities in these two parts, which needs further study. Another interesting note was the inflections in the blue region in the gynoecium and androecium and sepal fractions in Figure 2. This can also be explained by the relatively high content of Chl *a* in these two parts (Table 1). The absorption in the blue light region was caused by Chl *a*.

The peak at 3.2 min for the androecium/gynoecium in Figure 3b, which differed from the other spectra, was identified as β-carotene. In *A. amurensis*, AST exists as the *di*-esterified form (Figure 3a), which is consistent with the situation in *A. aestivalis* as reported by Kamata and Simpson [44]. Double esterification of AST with fatty acids added to 3 and 3′ hydroxyl groups of astaxanthin allow the AST to have more solubility and stability in the cellular environment. High di-ester AST content in the crude extract of *A. amurensis* flowers will also help to prolong the storage time of the pigment products. Esterification is also important to store the pigment in the hydrophobic environment. Interestingly, the proportions of free AST in the purple part of the petal and the androecium/gynoecium are significantly higher than that of the red area, which may mean less difficulty in getting highly pure free AST from these two parts without enzymatic hydrolysis.

The (3*S*,3′*S*) form has higher bioavailability and is more suitable for the health of humans [50]. Our result shows that (3*S*,3′*S*)-AST is the only optical isomer in *A. amurensis* (Figure 3c), which is consistent with the previous studies of *A. annua* [40], *A. aestivalis* [39], and *A. amurensis* [33], respectively.

In the androecium/ gynoecium, the ratio of all-*trans* AST was lowest; however, free, di-*cis*, and astacene had their highest values here as compared to their values in other flower parts (Figure 4). Little is known about the AST *cis-trans* isomerization pathway. To date, an epimerase for AST isomerization has not been identified. According to the results of studies conducted in humans [51] and rainbow trout [52], the in vivo isomerization of AST may lead to a different geometrical isomer composition. In *A. amurensis*, the mechanism(s) leading to the differences in AST composition between the *Adonis* flower components remains a question that needs to be investigated further. Given that animals cannot synthesize carotenoids *de novo*, AST in the shrimp must be obtained from food. When the white shrimp were fed using natural AST consisting of pure (3*S*,3′*S*)- isomer, the ratio of (3*S*,3′*S*): (3*S*,3′*R*): (3*R*,3′*R*) in the *L. vannamei* body (with the value 6:2:1) differed from that of its feed supplement [2]. In *A. aestivalis*, the cDNA encoding the enzyme that catalyzes the addition of the carbonyls has been identified, and the possible route to AST from β-carotene is clear, in which the addition of a carbonyl to carbon C_4_ involves a keto-enol tautomerization [37]. However, only a few studies have focused on the hydroxyl to C_3_ relating to the optical isomers in prawn [53,54,55]. In animals, the transformation mechanism of AST from (3*S*,3′*S*)- to (3*S*,3′*R*)- and/or (3*R*,3′*R*)-isomer is far from being clarified. We deduced that epimerases exist in the AST metabolic pathway both in animals and plants [25], which changes the initial optical isomer composition. A further comparative study performed on the AST pathways in animals and higher plants could provide more information about this unresolved issue.

### 3.2. Potential Uses of Adonis Flower AST

UVA can penetrate the dermis layer of the skin and damage the elastic fibers and collagen fibers, causing the skin to lose its elasticity. At the same time, it darkens the epidermis layer, while inflicting damage to the skin [56]. AST is commonly used in the cosmetics industry for its well-known antioxidant, anti-inflammatory, and antitumor properties [5]. The androecium and gynoecium of the *A. amurensis* flower appear to be a potential source of AST for cosmetic purposes if a process for sorting flower components can be made economically viable. In addition, AST has been approved as a dietary supplement and is widely used in the development of novel foods [31,57]. Improving the yield of AST from natural sources, such as *A. amurensis*, to satisfy the demands for human applications, especially the anti-ultraviolet agent, deserves further study. 

The function and application of specific carotenoids depend on their molecular structure [58]. From the thermodynamic point of view, the stability of all-*trans* AST is better than *cis* isomers but they may be isomerized from one form to another, and natural AST is easily oxidized and converted to astacene [28]. We have summarized the pigment content and isomer composition of most AST-containing organisms, in Table 2. *Cis* isomers together in *A. amurensis* flowers account for a higher proportion of AST (20.0%), in comparison with those of other natural sources. Compared with the all-*trans* isomer, *cis* AST, and especially 9-*cis* AST, has been shown to have higher antioxidant activity in vitro [28]. The highest concentration of *cis* isomers found so far is in the *A. amurensis* flower, which might make this a more suitable source for extraction of antioxidant material than other sources, e.g., *Haematococcus*, shrimp, and *P. rhodozyma*. There have been attempts to utilize AST from another *Adonis* species, *A. aestivalis*, as the pigmentation for fish [36,59], although its safety for humans has not been established.

### 3.3. Adonis Flower as a Promising Source of AST

The high production cost of natural AST is still the primary factor impacting the availability of this pigment. A previous study showed that the production cost of natural AST can be as low as €632/kg, which is lower than the cost for synthetic AST at €880/kg [45]. This encouraging price is not too surprising considering the low labor, land, and utility costs in China a decade ago. A recent assessment has shown that the production cost of natural AST is unable to compete with the synthetic alternative (as summarized in Table 3). The costs of natural AST in Livadeia and Amsterdam are €1536/kg and €6403/kg, respectively, both much higher than that of synthetic AST [32]. According to our results and published data, we estimate that the cost of producing natural AST from total *Adonis* flowers can be as low as €388–393/kg (Table 3), suggesting it is a very promising source for producing natural AST. As far as we know, the commercially available all-*trans* AST standard is a mixture of (3*S*,3′*S*), (3*S*,3′*R*) and (3*R*,3′*R*)-AST, and a pure all-*trans* isomer of (3*S*,3′*S*) AST is still lacking in the market. Our results suggest that Adonis flowers would be particularly suitable as raw material for obtaining pure all-*trans* (3*S*,3′*S*) AST.

## 4. Materials and Methods

### 4.1. Plant Materials and Reagents

**Figure 7:**
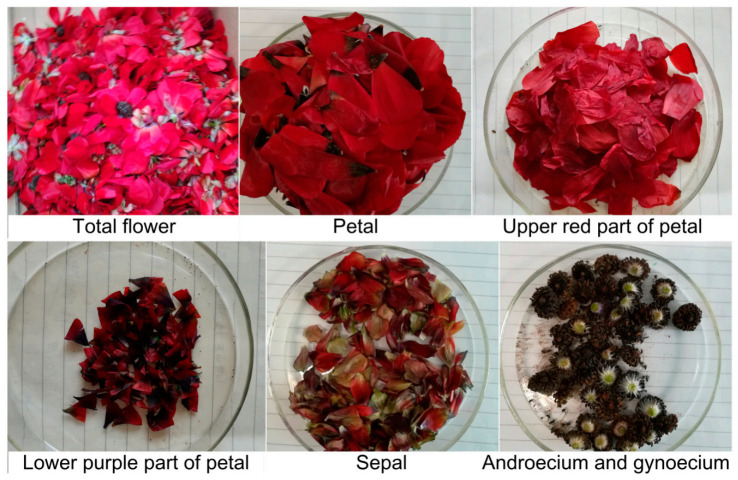
Samples of the different parts of the *Adonis amurensis* flower.

*A. amurensis* was cultivated by sowing in Shandong province, May 2017. Fresh flowers were handpicked from the plants and then sorted into different flower parts, i.e., total flower, the petal including the red spot and the purple spot, the sepal, and the remainder (mainly the combined androecium and gynoecium) (Figure 7). All materials were weighted, then stored in liquid nitrogen until further analysis.

An all-*trans* AST standard for AST quantification was obtained from Sigma Chemical Co., while cholesterol esterase for hydrolysis came from Wako Pure Chemical Industries, Ltd. HPLC-grade methanol, n-hexane, acetonitrile, methyl tert-butyl ether, and 2-propanol were obtained from Adamas Reagent Ltd. Acetone used as extracting reagent was pre-filtered through 0.22 μm GF/C filters (Whatman International Ltd., Maidstone, UK).

### 4.2. Preparation of Pigment Extracts from Adonis

Chl *a* and carotenoids from various flower parts were extracted by acetone using the modified method of Arnon [67] and Johnston et al. [68]. The tissue (about 0.5 g fresh weight) was placed in 10 mL centrifuge tubes in ice, then 6.0 mL of pre-cooled 100% acetone was added. The flower parts were blended, soaked for 30 min in acetone, and then homogenized in an ice-water bath. The pigment extracts were collected by centrifugation at 2500× *g* for 8 min at 4 °C. The cell debris was repeatedly extracted with the same solvent until it was colorless. All the pigment extracts for individual samples were merged into a 50 mL volumetric flask and then filtered using 0.22 μm GF/C filters (Whatman International Ltd., Maidstone, UK).

### 4.3. Quantification of Pigments

The acetone extracts were dehydrated with sodium sulfate. Then, the absorption spectra of pigment extracts were measured using a spectrophotometer (U-2900, HITACHI Co., Ltd., Tokyo, Japan). The optical path length was 10 mm. Chl *a*, Chl *b*, and total carotenoid concentrations of the acetone extract were calculated using the following equations [69]:

Chl *a* (%) = (12.7 × D_663 nm_ − 2.69 × D_645 nm_) × V/W (1)

Chl *b* (%) = (22.9 × D_645 nm_ − 4.68 × D_663 nm_) × V/W (2)

Carotenoids (%) = (D_480 nm_ − 0.634 × D_645 nm_ + 0.114×D_663 nm_) × V/2180/W (3)

where D_663 nm_, D_645 nm_, and D_480 nm_ represent the absorbance recorded at the indicated wavelengths, V is the diluted volume of the sample, W is the dry weight of the sample.

The moisture of the sample was calculated as:

Moisture (%) = 100 × (M_o_ − M_e_)/M_o_ (4)

where M_o_ is the wet weight of the fresh tissue; M_e_ is the dry weight of the biomass after drying for 24 h at 80 °C.

### 4.4. Identification of Carotenoids by HPLC

The hydrolysis of the carotenoid esters and the analysis of carotenoids followed the methods described in previous reports [69,70]. An Agilent 1200 HPLC system (Agilent Technologies Inc., Santa Clara, CA, USA) with a Luna Silica column (3 μm, 150 mm × 4.6 nm, Phenomenex, Torrance, CA, USA) was used for qualitative and quantitative analysis of carotenoids. Hydrolysis of astaxanthin was performed with cholesterol esterase for 60 min to remove esters according to our previous study [69] before identification and quantification by conventional HPLC. Identification of carotenoids without standards, such as di-*cis*-AST, astacene, and 15-*cis*-AST, was performed by comparing their spectra or retention times with those of published data [27,69,71,72,73]. Optical isomers of AST were further separated according to the method described by [29]. The levels of different optical isomers, i.e., (3*S*,3′*S*), (3*R*,3′R), and (3*S*,3′*R*)-AST, were calculated according to the peak areas and by comparing the retention time of sample peak to synthetic AST standard (CaroteNature, Lupsingen, Switzerland), respectively.

### 4.5. Preparation of the All-Trans Isomer of (3S,3′S)-AST

The all-*trans* isomer of (3*S*,3′*S*)-AST was prepared from the crude extracts of the total flower on an Agilent 1200 HPLC system (Agilent Technologies Inc., Santa Clara, CA, USA) with an InertSustain C18 column (5 μm, 250 mm × 10 mm, GL Science, Tokyo, Japan) [27]. The oily flowers extract is provided by the manufacturer settled in Weifang, China. The binary mobile phase consisted of A: 0.1% trifluoroacetic acid in water (*v*/*v*) and B: 95% methanol mixed with 5% acetonitrile (*v*/*v*). The solvent gradient was as follows: 0–5.8 min, 60–80% B; 5.8–32.6 min, 80–100% B; 32.6–35 min, 100% B; 35–47 min, 100–60% B; 47–50 min, 60% B. Peaks were detected at 470 nm. The flow rate was set at 3.0 mL/min. Under these conditions, the fraction containing the all-*trans* isomer of (3*S*,3′*S*)-AST eluted at a retention time of 34 to 35 min and was collected and dried under a nitrogen stream.

### 4.6. Statistics

SPSS 16.0 software was used for statistical analyses. One-way analysis of variance (*ANOVA*) and Tukey test (*p* < 0.05) was used to analyze differences for the multiple comparisons.

## 5. Conclusions

One optical isomer (3*S*, 3′*S*) of AST, with five geometrical isomers (all-*trans*, 9-*cis*, 13-*cis*, 15-*cis*, and di-*cis*) were observed in all parts of the flower, i.e., the petal including the red spot and the purple spot, the sepal, and the remainder (mainly the combined androecium and gynoecium). The highest carotenoid content was obtained from the red part of the petal (3.31%, dw). All-*trans* AST was the predominant geometrical isomer accounting for 72.5% of the total content of geometric isomers in total flower, followed by the 13-*cis* (9.8%), 9-*cis* (7.8%), di-*cis* (1.8%), and 15-*cis* (0.6%) isomers. The *cis*-AST extracted from the combined androecium and gynoecium can be used to prepare the anti-ultraviolet agents. The production cost of AST from *Adonis* flowers can be as low as €388–393/kg, which is far lower than that from *Haematococcus* culturing. Together with other factors such as the low technology requirement for plant culturing and harvesting, suggest *Adonis* has great potential as a resource for natural esterified (3*S*,3′*S*)-AST production.

## Figures and Tables

**Figure 6:**
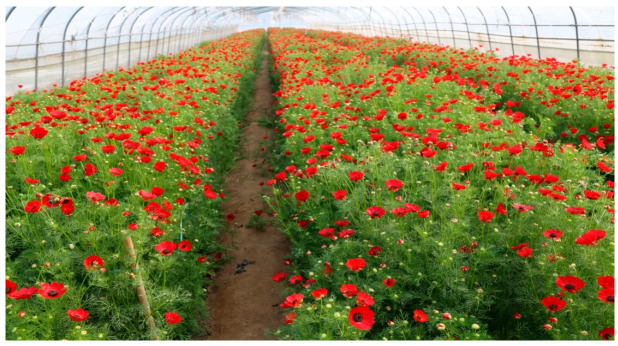
*Adonis* farming in a solar greenhouse covered by transparent plastic film.

**Table 1:** Pigments and moisture content in different parts of the *Adonis amurensis* flower.

Sample	Moisture (%)	Carotenoids (dw, %)	Chlorophyll a (dw, %)	Chlorophyll b (dw, %)
Total flower	69.13	1.31 ± 0.21	0.029 ± 0.003	0.021 ± 0.005
Petal	71.97	2.58 ± 0.10	*N. D.*	0.019 ± 0.005
Upper Red part of petal	67.72	3.31 ± 0.04	*N. D.*	0.027 ± 0.007
Lower Purple part of petal	73.85	1.21 ± 0.06	*N. D.*	0.010 ± 0.001
Sepal	67.70	0.32 ± 0.07	0.074 ± 0.013	0.029 ± 0.005
Androecium and gynoecium	69.42	0.14 ± 0.00	0.035 ± 0.002	0.018 ± 0.001

*N. D.* means not detected. dw means on biomass dry weight basis. Values are averages of four replicates ± standard error (SE).

**Table 2:** Distribution of astaxanthin in plants, aquatic animals, and zooplankton.

		Species	Astaxanthin Content	Proportion of Geometric Isomers (% of AST)					References
				All-*trans*	9-*cis*	13-*cis*	15-*cis*	di-*cis*	
Plant	Microalga	*Haematococcus pluvialis*	0.2–3.93 (% dw)	80–90	7–10	2–8	<1	N.D.	42
	Higher plant	*Adonis amurensis*	1.31 (% dw)	72	7.8	9.8	0.57	1.8	This study
Aquatic animals	Shrimp	*Litopenaeus vannamei*	1.9–271.4 (mg kg^−1^ww)	72–85	5–10	7–14	<2.5	<2	[2,23]
		*Penaeus monodon*	26.2–105.4 (mg kg^−1^ww)	70–80	6–15	2–17	2–4	<9	[23]
		*Fenneropenaeus chinensis*	1.9–137.7 (mg kg^−1^ww)	63–76	4–10	11–16	<9	<9	[23]
		*Exopalaemon carinicauda*	3.1–25.6 (mg kg^−1^ww)	72–92	3–9	5–17	<2	<3	[4,23]
		*Trachysalambria curvirostris*	11.0–106.0 (mg kg^−1^ww)	65–75	4–12	11–16	<4	4–7	[24]
	Crab	*Eriocheir sinensis*	12.9–343.4 (mg kg^−1^ww)	80–97	<6	1–8	1–13	N.D.	[60]
	Fish	*Oncorhychus mykiss*	2.1–4.3 (mg kg^−1^ww)	87–90	1–2	8–12	N.D.	N.D.	[61]
Zooplankton	Rotifer	*Brachionus plicatilis*	0.06–0.6 (mg g^−1^ww)	82–92	7–10	2–3	0.5–5	N.D.	[62]
	Cladoceran	*Moina macrocopa*	0.043–0.059 (mg g^−1^ww)	>90	N.D.	<10	N.D.	N.D.	Unpublished data
Yeast/Bacteria		*Phaffia rhodozyma*	11.4–13.4 (mg g^−1^ww)	70–78	2–3	15–21	4	2–3	[63]
		*Paracoccus carotinifaciens*	21.8 (mg g^−1^ww)	95.5	1.7	2.8	N.D.	N.D.	[64]

Note: N.D.: not detected; dw: dry weight; ww: wet weight. The data in shrimp are for the muscle, cephalothorax, and shell while the data in crab are for the ovaries and carapace.

**Table 3:** Productivity and production cost of natural astaxanthin using Adonis flower and *Haematococcus pluvialis*.

Resource	*Adonis* Flower	*Haematococcus pluvialis*		
Location	Inner Mongolia	Livadeia [32]	Amsterdam [32]	Shenzhen [45]
Production of biomass (kg/ha/year, dw)	1125 ^a^	18,280	6150	450
Astaxanthin content in the biomass	1.31%	---	---	2.50%
Production of astaxanthin (kg/ha/year)	14.74	426	143	11.25
Production costs of astaxanthin (€/kg)	388–393 ^b^	1536–1857	6403–6723	632

Note: a. the production was estimated according to the data provided by Huang [65]. b. the price was calculated according to the data provided by Li and Xiu [66], which covers the cost of mechanical operation, fertilizer, labor, watering, and land lease. The rent of film greenhouses (Figure 6) for planting flowers is €255/year.

## Data Availability

All relevant data can be found within the manuscript and its supporting materials.
